# Cognitive testing in 19 countries to refine WHO’s Sexual Health Assessment of Practices and Experiences

**DOI:** 10.2471/BLT.23.291162

**Published:** 2024-10-31

**Authors:** Erin C Hunter, Elizabeth Fine, Kirsten Black, Jacqueline Henriks, Fahmida Tofail, Chelsea Morroni, María Makuch, Kathleen Deering, Rocío Murad, Kwasi Torpey, Mamadou Dioulde Balde, Siswanto Agus Wilopo, Filippo Maria Nimbi, Beatrice Maina, Noor Ani Ahmad, Lalla Fatouma Traore, Thae Maung Maung, Adesola Olumide, Farina Abrejo, Dusita Phuengsamran, George William Ddaaki, Nicolás Brunet, Vanessa Brizuela, Lianne Gonsalves, on behalf of the Cognitive testing of a survey instrument to assess sexual practices, behaviours and health-related outcomes (CoTSIS) study group

**Affiliations:** a Department of Public Health Sciences, College of Behavior, Social and Health Sciences, Clemson University, Clemson, United States of America.; b School of Public Health, University of Sydney, Sydney, Australia.; c Discipline in Obstetrics and Gynaecology, University of Sydney, Sydney, Australia.; d School of Population Health, Curtin University, Perth, Australia.; e International Centre for Diarrhoeal Disease Research, Dhaka, Bangladesh.; f Botswana Sexual and Reproductive Health Initiative, Botswana Harvard Health Partnership, Gaborone, Botswana.; g Centro de Pesquisas em Saúde Reprodutiva de Campinas, Campinas, Brazil.; h Centre for Gender and Sexual Health Equity, Vancouver, Canada.; i Asociación Profamilia, Bogotá DC, Colombia.; j University of Ghana School of Public Health, Accra, Ghana.; k Center for Research in Reproductive Health in Guinea, Conakry, Guinea.; l Center for Reproductive Health, Universitas Gadjah Mada, Yogyakarta, Indonesia.; m Department of Dynamic and Clinical Psychology and Health Studies, Sapienza University of Rome, Rome, Italy.; n Sexual, Reproductive, Maternal, Newborn, Child and Adolescent Health Unit, African Population and Health Research Center, Nairobi, Kenya.; o Institute for Public Health, Ministry of Health Malaysia, Shah Alam, Malaysia.; p Faculté de Médecine et d’odontostomatologie, Université des Sciences, des Techniques et des Technologies de Bamako, Bamako, Mali.; q Yangon, Myanmar.; r College of Medicine, University of Ibadan, Ibadan, Nigeria.; s Department of Community Health Sciences, Aga Khan University, Karachi, Pakistan.; t Institute for Population and Social Research, Mahidol University, Nakhon Pathom, Thailand.; u Department of Social and Behavioral Sciences, Rakai Health Sciences Program, Kampala, Uganda.; v Institute of Health Psychology, Faculty of Psychology, Universidad de la República, Montevideo, Uruguay.; w Department of Sexual and Reproductive Health and Research, World Health Organization, Avenue Appia 20, 1211 Geneva 27, Switzerland.

## Abstract

**Objective:**

To refine a standard questionnaire on sexual practices, experiences and health-related outcomes to improve its cross-cultural applicability and interpretability. We aimed to explore participants’ willingness and ability to answer the draft questionnaire items, and determine whether items were interpreted as intended across diverse geographic and cultural environments.

**Methods:**

We conducted cognitive interviews (*n* = 645) in three iterative waves of data collection across 19 countries during March 2022–March 2023, with participants of diverse sex, gender, age and geography. Interviewers used a semi-structured field guide to elicit narratives from participants about their questionnaire item interpretation and response processes. Local study teams completed data analysis frameworks, and we conducted joint analysis meetings between data collection waves to identify question failures.

**Findings:**

Overall, we observed that participants were willing to respond to even the most sensitive questionnaire items on sexual biography and practices. We identified issues with the original questionnaire that (i) affected the willingness (acceptability) and ability (knowledge barriers) of participants to respond fully; and/or (ii) prevented participants from interpreting the questions as intended, including poor wording (source question error), cultural portability and very rarely translation error. Our revisions included adjusting item order and wording, adding preambles and implementation guidance, and removing items with limited cultural portability.

**Conclusion:**

We have demonstrated that a questionnaire exploring sexual practices, experiences and health-related outcomes can be comprehensible and acceptable by the general population in diverse global contexts, and have highlighted the importance of rigorous processes for the translation and cognitive testing of such a questionnaire.

## Résumé

**Objectif:**

Adapter un questionnaire standard sur les pratiques et expériences sexuelles ainsi que les résultats liés à la santé sexuelle, afin d'améliorer son intelligibilité et son applicabilité transculturelle. Nous souhaitions analyser la volonté des participants et leur capacité à répondre aux différentes thématiques abordées dans le projet de questionnaire, puis déterminer si certaines questions avaient été interprétées comme prévu selon les environnements géographiques et culturels.

**Méthodes:**

Nous avons mené des entretiens cognitifs (*n* = 645) répartis en trois périodes itératives de collecte de données dans 19 pays entre mars 2022 et mars 2023, avec des participants de sexes, genres, âges et origines différents. Les personnes chargées de l'entretien ont utilisé un guide pratique semi-structuré pour interroger les participants sur la manière dont ils ont interprété les questions et entrepris d'y répondre. Des équipes locales impliquées dans cette étude ont rempli des cadres d'analyse de données, puis nous avons organisé des réunions de réflexion conjointes entre les périodes de collecte de données afin de recenser les questions qui se sont soldées par un échec.

**Résultats:**

De manière générale, nous avons constaté que les participants étaient disposés à répondre au questionnaire, y compris aux thématiques les plus sensibles sur leur historique et leurs pratiques sexuelles. Nous avons identifié, dans le questionnaire initial, des problèmes qui (i) ont eu un impact sur la volonté (acceptabilité) et la capacité (connaissances insuffisantes) des participants à y répondre pleinement; et/ou (ii) ont empêché les participants d'interpréter les questions comme prévu, notamment en raison d'une mauvaise formulation (erreur dans la question source), d'une absence de transposition culturelle et, dans de très rares cas, d'une erreur de traduction. Dans le cadre de nos révisions, nous avons modifié l'ordre et la formulation des questions, ajouté des notes explicatives et un guide de mise en œuvre, mais aussi supprimé les questions difficiles à transposer dans d'autres contextes culturels.

**Conclusion:**

Nous avons montré qu'un questionnaire explorant les pratiques et expériences sexuelles ainsi que les résultats liés à la santé pouvait être compréhensible et acceptable pour l'ensemble de la population dans divers contextes à travers le monde. Nous avons également souligné l'importance d'établir des processus rigoureux de traduction et d'évaluation cognitive pour ce type de questionnaire.

## Resumen

**Objetivo:**

Ajustar un cuestionario estándar sobre las prácticas, las experiencias y los resultados relacionados con la salud sexual para mejorar su aplicabilidad e interpretabilidad transcultural. El objetivo consistía en explorar la disposición y la capacidad de los participantes para responder al borrador del cuestionario y determinar si las preguntas se interpretaban según lo previsto en diferentes entornos geográficos y culturales.

**Métodos:**

Se realizaron entrevistas cognitivas (n = 645) en tres rondas iterativas de recopilación de datos en 19 países durante marzo de 2022 y marzo de 2023, con participantes de diversos sexos, géneros, edades y regiones geográficas. Los entrevistadores utilizaron una guía de campo semiestructurada para obtener relatos de los participantes sobre sus procesos de interpretación y respuesta a las preguntas del cuestionario. Los equipos de estudio locales completaron los marcos de análisis de datos y se celebraron reuniones conjuntas de análisis entre las rondas de recopilación de datos para identificar fallos en las preguntas.

**Resultados:**

En general, se observó que los participantes estaban dispuestos a responder incluso a las preguntas más delicadas del cuestionario sobre biografía y prácticas en materia sexual. Se identificaron problemas con el cuestionario original que (i) afectaban a la disposición (aceptabilidad) y la capacidad (barreras de conocimiento) de los participantes para responder plenamente; o (ii) evitaban que los participantes interpretaran las preguntas según lo previsto, incluida una redacción deficiente (error en la pregunta original), portabilidad cultural y, muy raramente, error de traducción. Las revisiones incluyeron el ajuste del orden y la redacción de las preguntas, la adición de preámbulos y orientaciones de implementación, y la eliminación de preguntas con una portabilidad cultural limitada.

**Conclusión:**

Se ha demostrado que un cuestionario que explora las prácticas, las experiencias y los resultados relacionados con la salud sexual puede ser comprensible y aceptable para la población general en diversos contextos mundiales. Además, se ha destacado la importancia de contar con procesos rigurosos para la traducción y las pruebas cognitivas de un cuestionario de este tipo.

## ملخص

**الغرض:**

تنقيح الاستبيان القياسي حول الممارسات والتجارب الجنسية، والنتائج المتعلقة بالصحة، لتحسين قابلية تطبيقه وتفسيره عبر الثقافات. كنا نهدف إلى استكشاف رغبة المشاركين وقدرتهم على الإجابة على بنود الاستبيان الأولي، وتحديد ما إذا كانت البنود قد تم تفسيرها على النحو المقصود عبر بيئات جغرافية وثقافية متنوعة.

**الطريقة:**

قمنا بإجراء مقابلات معرفية (ن = 645) في ثلاث موجات متكررة لجمع البيانات عبر 19 دولة من مارس/آذار 2022 إلى مارس/آذار 2023، مع مشاركين من أجناس، وأنواع، وأعمار متنوعة، ومناطق جغرافية مختلفة. استخدم القائمون بالمقابلات دليلاً ميدانيًا شبه منظم لاستخلاص أفكار من المشاركين بخصوص عمليات تفسير بنود الاستبيان والاستجابة لها. أكملت فرق الدراسة المحلية أطر عمل تحليل البيانات، وقمنا بإجراء اجتماعات للتحليل المشترك بين موجات جمع البيانات، لتحديد الإخفاقات في الأسئلة.

**النتائج:**

بشكل عام، لاحظنا أن المشاركين كانوا يرغبون في الرد حتى على أكثر بنود الاستبيان حساسية حول السيرة والممارسات الجنسية. قمنا بتحديد مشكلات في الاستبيان الأصلي والتي (أ) أثرت على رغبة (قبول) وقدرة (حواجز المعرفة) المشاركين على الاستجابة الكاملة؛ و/أو (ب) منعت المشاركين من تفسير الأسئلة على النحو المقصود، بما في ذلك الصياغة الرديئة (خطأ في أصل السؤال)، وقابلية النقل الثقافي، ونادرًا جدًا خطأ الترجمة. تضمنت مراجعاتنا تعديل ترتيب البنود وصياغتها، وإضافة مقدمات وتوجهات للتنفيذ، وإزالة البنود ذات قابلية النقل الثقافي المحدودة.

**الاستنتاج:**

لقد أوضحنا أن الاستبيان الذي يستكشف الممارسات الجنسية، والتجارب، والنتائج المتعلقة بالصحة، يمكن أن يكون مفهومًا ومقبولًا من جانب عامة السكان في أوضاع عالمية متنوعة، وقمنا بالتركيز على أهمية العمليات الجادة لترجمة مثل هذا الاستبيان واختباره معرفيًا.

## 摘要

**目的:**

完善用于了解性行为、性经历和性健康相关结果的标准调查问卷,以提高其跨文化适用性和可解读性。我们旨在探讨参与者回答调查问卷(草稿)中题目的意愿和能力,并确定在不同的地理和文化环境中人们是否会以预期方式解读这些题目。

**方法:**

在 2022 年 3 月至 2023 年 3 月期间,我们在 19 个国家反复开展了三波认知访谈 (*n* = 645) 以进行数据搜集,其中参与者涵盖了具有不同生理性别、社会性别、年龄和来自不同地理位置的人们。访谈员借助一个半结构化的现场指南来引导参与者,让他们就调查问卷的题目解读以及回答过程发表看法。当地研究团队构建了数据分析框架,我们利用两波数据收集活动之间的间隔时间举行了联合分析会议,以找出设计失败的问题。

**结果:**

总体而言,我们观察到参与者甚至愿意回答调查问卷中最敏感的性经历和性行为相关题目。我们发现原始调查问卷存在以下方面的问题:(i) 影响参与者充分回答的意愿(可接受性)和能力(知识障碍);和/或 (ii) 妨碍参与者以预期方式解读问题,包括措辞不当(源问题错误)、文化可移植性和极少数的翻译错误。我们的修订工作包括调整题目顺序和措辞、增加开场白和实施指导,以及删除文化可移植性有限的题目。

**结论:**

研究表明,在全球多元化的背景下,一份探讨性行为、性经历和性健康相关结果的调查问卷是可以为普通民众所解读和接受的,而对此类调查问卷的翻译和认知测试需要遵循严格的流程,这一点非常重要。

## Резюме

**Цель:**

Усовершенствовать стандартную анкету о сексуальном поведении, опыте и результатах, связанных со здоровьем, с целью повышения ее межкультурной применимости и интерпретируемости. Перед авторами стояла задача изучить готовность и способность участников отвечать на вопросы проекта анкеты, а также определить правильность интерпретации пунктов в различных географических и культурных средах.

**Методы:**

С марта 2022 года по март 2023 года были проведены когнитивные интервью (*n* = 645) в трех итеративных волнах сбора данных в 19 странах с участниками разного пола, гендера, возраста и географической принадлежности. Интервьюеры использовали полуструктурированное руководство для получения от участников пояснений о том, как они интерпретировали пункты анкеты и отвечали на них. Региональные исследовательские группы составили схемы анализа данных, после чего были проведены совместные аналитические совещания между волнами сбора данных для выявления неудачных вопросов.

**Результаты:**

В целом отмечено, что участники охотно отвечали даже на самые деликатные пункты анкеты, касающиеся сексуальной биографии и поведения. Были выявлены проблемы с исходной анкетой, которые (i) влияли на готовность (приемлемость) и способность (барьеры знаний) участников отвечать в полном объеме и/или (ii) не позволяли участникам интерпретировать вопросы должным образом, включая неудачные формулировки (ошибка исходного вопроса), разницу культурных подходов и очень редко ошибки перевода. Внесенные изменения включали корректировку порядка пунктов и формулировок, добавление преамбул и руководства по применению, а также удаление пунктов, воспринимаемых неоднозначно из-за разницы культурных подходов.

**Вывод:**

В результате было показано, что анкета, посвященная изучению сексуального поведения, опыта и последствий для здоровья, может быть понятна и приемлема для населения в различных глобальных контекстах, а также подчеркнута важность строгих процессов перевода и когнитивного тестирования такой анкеты.

## Introduction

Despite decades of research, programming and investment into improving sexual and reproductive health and rights outcomes, there has been inadequate attention paid to sexual activity.^1^^,^^2^ Information, education and services can better meet the sexual and reproductive health and rights needs of people if the practices underpinning these, along with their broader social contexts, are better understood. However, research on practices related to sexual health and on sexuality remains sensitive, marginalized and neglected in many parts of the world.^2^ Although some high-income countries have conducted national surveys^3^^,^^4^ yielding strong population-level data, only a few studies have included data from more than one country. These multicountry studies^5^^–^^7^ have been limited in the scope of practices assessed and populations included, which has resulted in fragmented global data that limits comparative research.^8^ Comparable, cross-national, population-representative data can help identify differences in health outcomes and provide a better understanding of social norms related to gender, sexuality and sexual practices; such an enhanced understanding is necessary to improve health equity and ensure that health services meet the needs of the populations they serve.

To address this gap, the United Nations Development Programme, United Nations Population Fund, United Nations Children’s Fund, World Health Organization (WHO) and World Bank Special Programme of Research, Development and Research Training in Human Reproduction (the Human Reproduction Programme) initiated a multiphase consultative process at WHO headquarters in Geneva in 2019. The aim of this process was to develop a standard survey questionnaire that would enable researchers to collect data in the general population on sexual practices, experiences and health-related outcomes, facilitating comparisons within and between countries. Details of the consultative process that resulted in a draft questionnaire are provided elsewhere.^9^^,^^10^

In this paper we present the main results of the Cognitive testing of a survey instrument to assess sexual practices, behaviours and health-related outcomes (CoTSIS) study that was implemented during 2021–2023 to pretest and refine the draft questionnaire. Cognitive interviewing is a qualitative method that enables an exploration of the considerations made by study participants as they hear, process and respond to survey questions.^11^ This type of interviewing can help to identify sources of response errors in a quantitative survey during pretesting, and guide questionnaire revision to improve validity before fielding the full survey; cognitive interviewing is therefore an important, yet too often neglected, step in survey questionnaire development. Cognitive interviewing studies in global health have found instances of extensive mismatch between the intent of survey questions and the interpretations of participants, which can severely compromise the validity of a survey if not addressed.^12^

Our aim was to produce a refined questionnaire that would be interpretable and applicable to the general population (age ≥ 15 years) in diverse geographic and cultural environments. Cognitive interviewing was a particularly critical component of our survey questionnaire development because of its intended purpose of facilitating comparative research on a sensitive topic across global contexts. Our cognitive interviewing study aimed to explore the willingness and ability of participants to answer the draft questionnaire items about their sexual practices, experiences and health-related outcomes, and whether participants interpreted the questionnaire items as intended.

## Methods

### Study design and team training

**Fig. 1:**
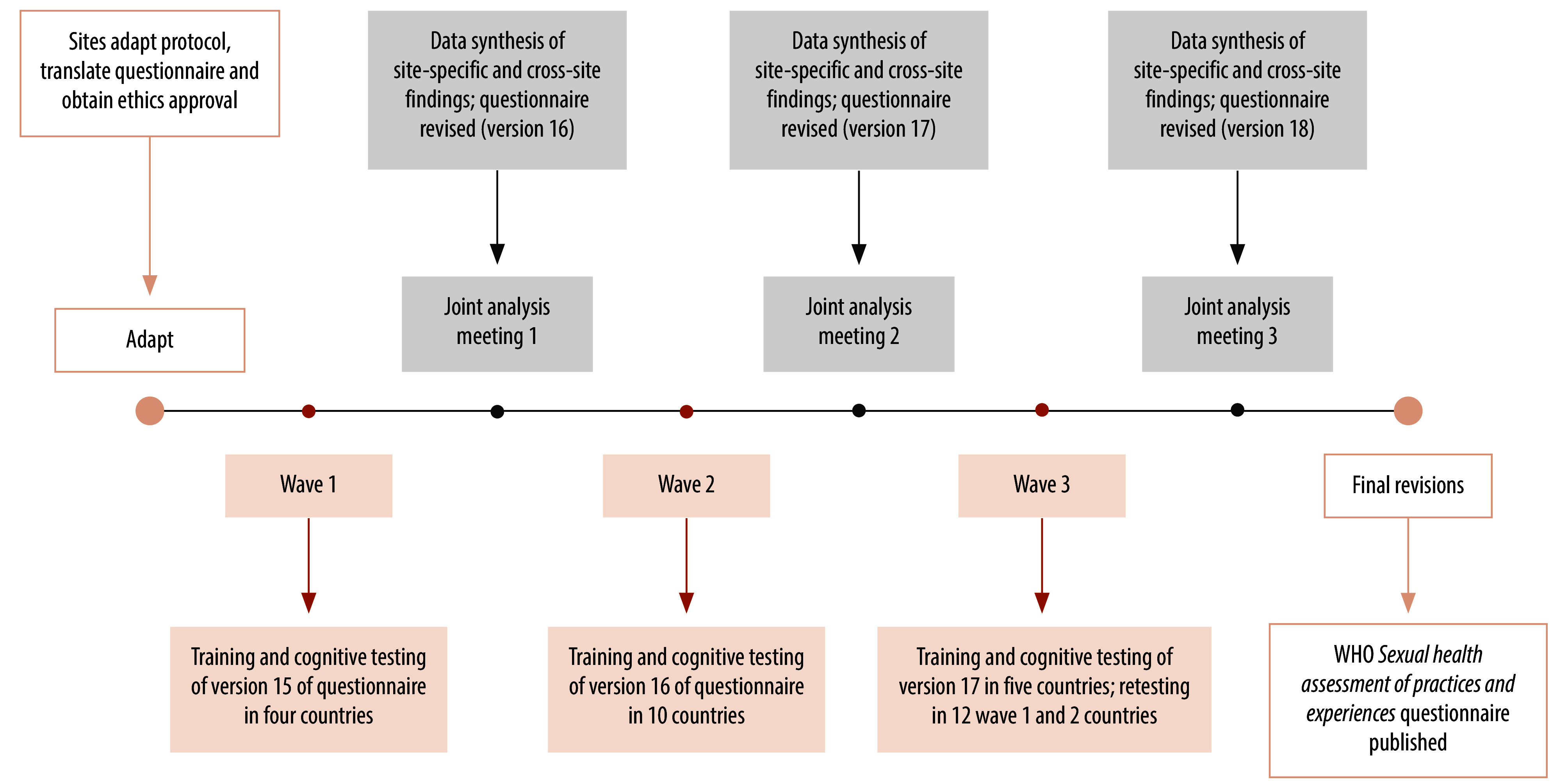
Iterative cognitive interviewing study design to revise questionnaire on sexual practices, experiences and health-related outcomes

Study methods are described in the protocol of our cognitive interviewing study.^13^ We selected research collaborators from 19 countries (Table 1; online repository)^14^ who responded to an open call from the Human Reproduction Programme Alliance^15^^,^^16^ to implement the study. Study sites included low-, middle- and high-income countries across all WHO regions. We divided sites into groups to complete training and data collection in three consecutive waves, allowing for iterative analysis and refinement of the questionnaire (Fig. 1). With technical support from an external study steering group,^14^ we coordinated this global study from WHO headquarters.

**Table 1:** Summary of participant characteristics in the cognitive interviewing study for refining the questionnaire on sexual practices, experiences and health-related outcomes

Country (language)	Total no. interviews (in rural settings)	Sex assigned at birth			Age, years					Highest level of education,^a^ no.		
		Male	Female		15–19	20–24	25–59	> 60		Elementary or less	Some secondary	Some tertiary
Australia (English)	35 (6)	12	23		1	0	31	3		0	0	34
Bangladesh (Bangla)	39 (0)	21	18		9	11	11	8		11	23	5
Botswana (Setswana)	14 (0)	4	10		1	5	8	0		2	12	0
Brazil (Portuguese)	40 (2)	19	21		8	9	15	8		8	16	16
Canada (English)	20 (2)	7	13		4	5	7	4		2	3	15
Colombia (Spanish)	42 (10)	21	21		10	9	13	10		2	25	15
Ghana (English)	34 (11)	16	18		8	7	11	8		1	7	26
Guinea (French)	34 (10)	17	17		9	9	10	6		0	17	17
Indonesia (Indonesian)	32 (12)	17	15		6	6	13	7		2	9	20
Italy (Italian)	40 (1)	21	19		8	12	16	4		0	16	24
Kenya (Swahili)	44 (19)	21	23		6	10	20	8		8	22	14
Malaysia (Malay)	24 (6)	12	12		2	4	17	1		0	1	23
Mali (Bambara)	42 (19)	20	22		10	7	17	8		22	9	11
Myanmar (Burmese)	34 (0)	16	18		7	8	13	6		6	24	4
Nigeria (English)	42 (21)	20	22		7	10	21	4		2	11	29
Pakistan (Urdu)	48 (4)	23	25		5	12	23	8		4	25	19
Thailand (Thai)	42 (20)	21	21		8	10	20	4		3	16	23
Uganda (Luganda)	24 (13)	12	12		6	5	8	5		16	6	2
Uruguay (Spanish)	15 (0)	1	14		1	4	10	0		0	0	15
**Total**	**645**	**300**	**345 **		**117 **	**141 **	**285 **	**102 **		**89 **	**249 **	**305 **

^a^ Information on highest level of education is missing for one participant from Australia and one participant from Indonesia; level of education was reported differently between sites according to local conventions.

Each country team included researchers with experience in qualitative interviewing and sexual and reproductive health. Study protocol training covered study procedures, cognitive interviewing methods, participant distress and safety, reflexivity and interviewer well-being. Training comprised videos, guided activities, role-plays and two half-day interactive online sessions focused on skills practice. Teams had access to additional training, including values clarification and attitude transformation workshops offered through the Human Reproduction Programme Alliance hub at the African Population Health Research Center, Nairobi, Kenya.^15^

### Data collection

**Fig. 2:**
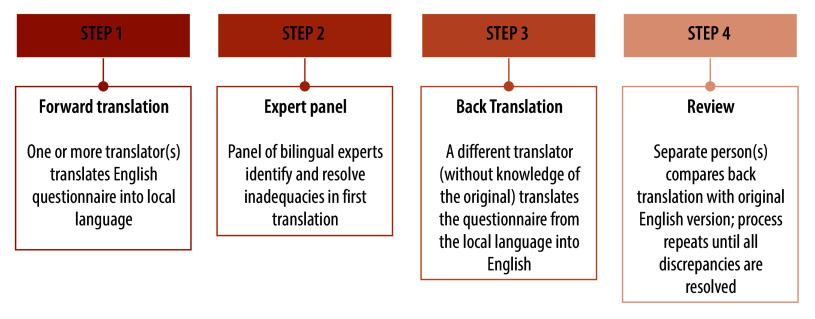
Example of the (site-dependent) translation process for the cognitive interviewing study to revise questionnaire on sexual practices, experiences and health-related outcomes

Data collection occurred during March 2022–March 2023. Implementation at each site began with the development of a local-language version of the English source questionnaire according to rigorous translation plans (Fig. 2) that met minimum standards (Box 1).

Box 1 **Minimum standards applied in the cognitive interviewing study to the translation process**

- The translators had full professional proficiency in English and target language (spoken and written).
- Separate individuals performed forward translation(s) and back translation(s).
- Initial forward and back translations were performed independently of each other, then compared.
- An expert panel (including e.g. translators, study team members, sexual health experts and researchers experienced in instrument development and translation) was involved to adjudicate.
- We aimed to achieve conceptual equivalence of words/phrases, as opposed to literal word-for-word translations.
- We used the language of the general population with basic reading level (no jargon).

Participant recruitment methods varied by country,^14^ ranging from social media advertisements to in-person outreach. Potential participants were informed that WHO was developing a questionnaire about sexual health that could be used in different countries. Interviewers explained that we wanted to trial these questions and explore how participants decided on responses, so that we could gauge their willingness and ability to answer as well as their understanding of the questions.

Participants were purposively selected to ensure inclusion across sexes, age groups, geography, identities and experiences (e.g. including those with many sexual experiences within the past year and those without any sexual experiences, as well as participants across the diversity of sexual orientations and gender identities).^13^ Snowball sampling was used when needed to increase the recruitment of older adults. Sampling decisions were guided by the goal of achieving theoretical saturation.^13^

Cognitive interviewing using a semi-structured field guide was used to elicit narratives from participants about the processes they went through in interpreting and responding to the questionnaire items. The initial version of the draft questionnaire contained six modules: (A) sociodemographics and health (nine items); (B) sexual health outcomes (14 items); (C) sexual biography (11 items); (D) sexual practices (18 items); (E) social perceptions and beliefs (13 items); and (F) identity and rights (10 items).^13^ The cognitive interview field guide included the draft questionnaire and suggested probing questions (Box 2), although interviewers also probed spontaneously to elicit rich descriptions.

Box 2 **Example probing questions used in the cognitive interviewing study for refining the questionnaire on sexual practices, experiences and health-related outcomes**

**Approach to probing**

- Interviewers had scripted, suggested probes in the field guide to use during cognitive interviews, but also used their own spontaneous probing questions as needed to elicit rich descriptions from participants.
- Interviewers primarily probed concurrently, meaning they asked probing questions immediately after participants responded to each individual questionnaire item. Some retrospective probing questions were then asked at the end of each module to understand participants’ experience with the module as a whole.^17^
- At a few sites, participants completed items in the self-administered modules on their own and then responded to probing questions from the interviewer retrospectively (see online repository).^14^

**Example probing questions**

What did you think this question was asking you?

How did you feel about being asked this question?

What did [X term/phrase] mean to you?

How did you calculate your response to this question?

How easy or difficult was this to remember and answer?

What made you choose the answer you did?

Because of the coronavirus disease 2019 (COVID-19) pandemic, remote data collection via teleconference was necessary in some countries. Where in-person cognitive interviews were feasible, interviewers conducted these one-to-one in locations that participants felt were adequately private and comfortable (e.g. private office or home), observing local COVID-19 protocols. Study teams audio-recorded interviews and transcribed these in the local language, using the fieldnotes of interviewers about participants’ non-verbal communications to enrich the data. Participant reimbursement policies adhered to local conventions.

### Data analysis

Following each interview, study teams synthesized raw data from audio recordings and field notes into English-language summaries across each item by completing a data analysis framework in an electronic spreadsheet (see online repository).^14^ Study teams held debriefing meetings with the global study coordinators after their first two interviews to troubleshoot early issues, and continued regular internal debriefing meetings throughout data collection to aid participant selection decisions and iterative data analysis.

Study teams compared findings for each item across all participants within their site, and then shared their completed data analysis framework and a summary of key findings for comparison at the global level. A global synthesis identified patterns in interpretations and question failures across all sites and subgroups of participants, which were then discussed during a joint analysis meeting after each data collection wave. We revised the draft questionnaire after each joint analysis meeting to clarify constructs, improve item interpretability and enhance user experience before the next data collection wave (Fig. 1).

### Ethical considerations

The master protocol (ERC.0003501) and site-specific protocols received approval from the WHO Ethics Review Committee and local or national boards (see online repository).^14^ All study participants provided informed consent. Country-specific adaptations included type of consent, whether waivers of guardian consent for adolescents were allowable, and locally tailored protections of participants from legal or social risks that could arise from involvement in the study.^14^ Unique identifiers replaced personally identifiable information about study participants.

## Results

Here we describe high-level patterns of the results that contributed to questionnaire revisions; detailed country- and regional-level results, and item-specific findings disaggregated by participant subgroups, will be published separately. 

Study teams conducted a total of 645 cognitive interviews, lasting an average of 84 minutes. The ages of participants ranged from 15 to 86 years (mean: 34.5 years; standard deviation: 16.6). Table 1 provides a summary of information about the study participants. 

When discussing responses during joint analysis meetings, we refined the modules to (A) personal information and health (eight items); (B) sexual health outcomes (15 items); (C) sexual biography (12 items); (D) sexual practices (21 items); (E) social perceptions/beliefs (13 items); and (F) sociodemographics (six items). We found failures in questionnaire items previously used in other surveys as well as in items that had been newly developed specifically for our questionnaire; Table 2 provides illustrative examples of item revisions made in response to question failures. Revisions included re-ordering items, revising skip patterns, changing item wording and response options, splitting complex questions, removing items, adding preambles and providing implementation guidance notes. We provide a longer list of examples in the online repository.^14^

**Table 2:** Examples of revisions made to items in the questionnaire on sexual practices, experiences and health-related outcomes during the cognitive interviewing study

Original item	Error type and description	Final item	Summary of revisions
A1. At birth, were you described as male; female; or intersex, undetermined, or another sex?	Source question issue: original phrasing of the item caused minor confusion across sites Knowledge barrier and source question issue: “intersex” was not well understood and current best practice suggests asking about intersex variations separately from sex assigned at birth	A1. At birth, was your sex recorded as male, female, or another term (please specify)? A1 alternative: What was your sex assigned at birth?	Modified question stem to clarify the construct being measured. Added translation/adaptation note to use alternative version where term “sex assigned at birth” is well understood. Modified response options to remove “intersex, undetermined or another sex” (separate intersex item added, but ultimately removed after testing because of extensive response errors)
A2. Today, do you think of yourself as: man/boy, woman/girl, or in another way (please specify)?	Source question issue: although most participants understood this question to be about current gender identity, some were confused by the combination of “man/boy” and “woman/girl” (e.g. some participants in Nigeria thought they were being asked if they considered themselves mature or grown-up)	A2. Today, do you think of yourself as: man/male, woman/female, or in another way (please specify)?	Modified response options to remove boy and girl to reduce confusion, because the item was not about age or maturity. Translation and adaptation note added to encourage researchers to use the most commonly understood terms referring to gender identities in their context
A4. Are you at present single, married, separated but still legally married, divorced, or widowed?	Source question issue: item was intended to assess marital and civil status yet participants across multiple sites often interpreted it to be about relationship status more broadly, and did not want to use the “single” response option if in a long-term relationship	F1. What is your marital status? Never married, Married, Separated but still legally married, Divorced, Widowed, Prefer not to say	Modified question stem to clearly ask the construct being measured; modified “single” response option to “never married” to ask for marital status more clearly. Item moved to demographic questions (Module F). Translation and adaptation note added to encourage localization of response options, as appropriate
B1V2. To the best of your knowledge, how many times have you gotten someone pregnant to date?	Source question issue: participants often did not consider pregnancies that ended in abortion, miscarriage or stillbirth in their responses	B1V2. To the best of your knowledge, how many times have you gotten someone pregnant to date, including any pregnancies that did not end in a live birth?	Added clarifying phrase to prompt participants to consider pregnancies which did not end in a live birth, consistent with item intent
B10. Aside from HIV, when, if ever, were you last tested for any sexually transmitted infections (STIs) (e.g. gonorrhoea, chlamydia, syphilis, herpes, trichomoniasis, etc)? Was it in the last year, more than 1 year ago, never, don’t know/don’t remember or prefer not to say?	Source question issue: minor confusion regarding the intended timeframe of “in the last year.” Knowledge barrier: examples in the body of the item were found to be confusing and distracting to participants; however, without examples, there were clear knowledge barriers to answering the question	B11. Aside from HIV, when, if ever, were you last tested for any sexually transmitted infections (STIs)? Was it within the last year, more than 1 year ago or never? Interviewer note: If a participant does not understand the term “sexually transmitted infection (STI)” when first asked the question, you can provide a definition: There are infections that are transmitted through sexual contact, including vaginal, anal and oral sex. These can include chlamydia, gonorrhoea, herpes, syphilis (insert local terms for common STIs here).	Removed examples and added an interviewer note to prompt interviewers to assist participants, as needed. Minor revision to response option “in the last year” to read “within the last year” and translation and adaptation note added to clarify that this option is meant to capture the preceding 12 months before the interview
B12. Currently, in your everyday life (i.e. at work, on the street, at home), how safe do you feel from sexual assault? Not at all safe, Somewhat unsafe, Neither safe or unsafe, Somewhat safe, Completely safe, or It varies or unsure	Source question issue: participants were unable to address safety in their home and outside their home with a single response; “neither safe or unsafe” and “it varies” responses were not well understood and were used similarly to other response options (e.g. “somewhat safe” and “somewhat unsafe”)	B13.1. At home, how safe do you typically feel from sexual assault: not at all safe, somewhat unsafe, somewhat safe, completely safe? Don’t know or prefer not to say B13.2. As above, but “At home” changed to “Outside your home, for example at work or on the street”	Simplified question (split into two) to capture feelings of safety in the home (B13.1) and outside the home (B13.2); revised response options to remove “neither safe or unsafe” and “it varies”
D6. The most recent time you had sex, what did you consider the ethnicity of the person you had sex with to be?	Acceptability: some considered the item offensive Cultural portability: “ethnicity” as a construct was understood and interpreted differently across settings; many participants were unable to answer accurately	Item removed	Item removed
E12. It is okay for a women [to have an abortion/terminate a pregnancy] if she does not want to have a child: Strongly agree, Agree, Disagree, Strongly disagree or Prefer not to answer	Source question issue: original item format (Likert scale) was difficult for participants as many wished to express more nuanced views. Acceptability: gender was not relevant for the measurement aim. Translation error: “okay” was ambiguous when translated from the English into other languages during testing	E12. Which of these statements is closest to your personal view? It is okay for someone to have an abortion/terminate a pregnancy for any reason if they want to; It is only okay for someone to have an abortion/terminate a pregnancy under certain circumstances; It is always wrong for someone to have an abortion/terminate a pregnancy, regardless of circumstances; Prefer not to say	Revised item format, item stem and response options to better reflect nuanced views; gendered language was removed. Translation and adaptation note added to clarify the meaning of “okay”

HIV: human immunodeficiency virus; STI: sexually transmitted infection.

We identified issues with the questionnaire in its original form that (i) affected the willingness (acceptability) and ability (knowledge barriers) of participants to respond fully; and/or (ii) prevented participants from interpreting the questions as intended, including poor wording (source question error), cultural portability and very rarely translation error. 

### Acceptability

Overall, participants across country sites were willing to respond to the questionnaire items, even the most sensitive modules on sexual biography and sexual practices. While noting that questions were indeed sensitive, participants often remarked on the importance of such research. Others noted that they appreciated the opportunity to discuss these issues because they had rarely or never spoken about them with others. Exceptions included instances where a few participants felt certain items were too exclusionary, or otherwise perceived as irrelevant to them personally or out of alignment with their values. For example, a few participants did not respond to items about particular sexual practices that they were opposed to or uninterested in, rather than selecting the “never” response option. This outcome was more common in items referring to various forms of anal and oral sex.

Some participants across a few sites voiced frustration over the gender-binary nature of items in Module E (social perceptions and beliefs), noting it “…doesn’t make room for people like me, as a nonbinary person” (age 38, Canada). Discussion on this issue during all three joint analysis meetings led to a decision to make items in this module gender neutral where possible, for example when asking about perceptions around a given practice (e.g. “It is okay for someone to use a contraceptive method/family planning to avoid or delay pregnancy” instead of “It is okay for a woman to use a modern contraceptive method/family planning (e.g. birth control/oral contraceptive pills, injection, implants, loop or coil (IUD), condoms, etc.) to avoid or delay pregnancy if she wishes”). Exceptions to this change were for items that purposefully aimed to assess perceptions about a specific gender, for example, “A woman has the right to say ‘no’ to sex if she does not want it.” We added implementation guidance to instruct researchers to consider whether adding specifications – such as whether items about women refer to transgender women – would be more acceptable in their context. Cognitive interview data also suggested that some participants might have chosen certain responses in this module to appear more favourably to the interviewer. Upon recommendation from multiple study sites, the interviewer notes for the final questionnaire suggest that this module be self-administered to reduce social desirability bias.

Because participants who had experienced non-consensual sex were unsure whether to include such experiences when responding to various items (e.g. age at first sex, number of sexual partners and satisfaction with their sex life), we made multiple revisions to improve the survey experience for such participants. We addressed these issues by reordering items to identify earlier in the interview whether participants had non-consensual experiences, and providing clearer preambles and screening questions to enable participants to opt out of sections about non-consensual experiences. We also created alternative forms of questions that were more appropriately worded for those choosing to report experiences that were non-consensual.

### Knowledge barriers

Knowledge barriers caused participants difficulty in responding to a few items. An item in Module A about whether participants have intersex variations performed poorly in most settings. Despite including a description of intersex variations, many participants did not understand what was being asked, resulting in a nonresponse or misclassification. For example, many participants described choosing the response option “unsure” because they did not understand what they were being asked, although the “unsure” response option was intended to indicate a participant’s uncertainty around whether or not they had an intersex variation. Participants across sites often interpreted the question as being about gender identity or expression: “…people actually say even though I am a guy sometimes I speak and act as a lady” (age 23 years, Ghana). We eliminated the item on intersex variations from the final questionnaire because it consistently generated poor-quality data. However, we did add implementation guidance to suggest considering its use in settings with greater awareness of intersex. 

Knowledge barriers also contributed to issues with items about sexually transmitted infections in Module B (Table 2). Because some participants did not understand what a sexually transmitted infection was, or were not familiar with the names of specific types, we added a definition in interviewer notes and suggested prompting with the names of specific types only if necessary. 

### Source question issues

Discordance between the measurement aims of items and interpretations of participants most often stemmed from imprecise wording in the source questionnaire. We resolved many issues easily by adding words or phrases to items and response options. For example, participants failed to account for pregnancies ending in spontaneous or induced abortions (e.g. “I counted when she delivered a child”; age 25 years, Kenya) when asked the item “To the best of your knowledge, how many times have you gotten someone pregnant to date?” in Module B. Adding “…including any pregnancies that did not result in a live birth” addressed the issue. 

Other items required more substantial modifications to clarify constructs being assessed. For instance, the item “Are you at present single, married, separated but legally married, divorced or widowed?” (originally in Module A) had high nonresponse at multiple sites because participants interpreted it as about relationship status rather than marital or registered civil status. As a result, participants who were unmarried but were dating or in long-term relationships did not find “single” to be a suitable response (e.g. “I wouldn’t consider myself any of those, I would consider myself in a relationship”; age 37 years, Australia). After testing multiple iterations, the final version (now in Module F) explicitly states the intended construct: “What is your marital status?” with the response options “never married, married, separated but still legally married, divorced, widowed or prefer not to say,” with a translation and adaptation note instructing interviewers to adapt this item to include other legal civil designations (e.g. civil union) if applicable.

Some items asked about more than one issue while only allowing a single answer. For example, participants struggled to respond to the Module B item “Currently, in your everyday life (i.e. at work, on the street, at home), how safe do you feel from sexual assault?” because of the large variation between their sense of safety outside the home compared with inside the home. Some participants chose to prioritize one location when considering their response, others tried to average across locations and others just chose the response option “It varies or unsure.” This issue was addressed by splitting the single item into two separate items, one for at home and the other for not at home (Table 2).

Likert scale response options contributed to notable measurement error in Module E (social perceptions and beliefs). For example, an item asking participants to indicate whether they strongly agree, agree, disagree, strongly disagree or prefer not to respond to the statement “It is okay for a woman to have an abortion/terminate a pregnancy if she does not want to have a child” generated noisy data. Many participants across sites described how their opinions were dependent on circumstances, for example, whether the person was married, there were medical issues or they had been sexually assaulted. Participants expressing these same opinions chose vastly different responses across the Likert scale, cautioning against drawing conclusions from the quantitative data generated by this item. After multiple iterations to improve clarity and gender inclusivity, the final version was entirely restructured (Table 2), as were several other items in the module (e.g. who should make the decision about someone having an abortion; whether men or women naturally have more sexual needs; whether sex between two consenting adults of the same sex is wrong; and sex education in school).

### Cultural portability

A few items were removed from the questionnaire because of diverse conceptualizations of the constructs intended for measurement, making standardization infeasible. For instance, an item in an early version of the questionnaire (“The most recent time you had sex, what did you consider the ethnicity of the person you had sex with to be?”) was problematic at nearly every site. Interpretations of ethnicity varied widely, with participants in some settings emphasizing tribal affiliation and others identifying by skin colour. In more ethnically homogenous populations, participants of the predominant ethnic group struggled to understand what they were being asked, as they were more familiar with identifying simply by nationality or by religion. Participants questioned the relevance of the information and some even considered the question to be offensive or “racist.” In countries where ethnic conflict is common, this question was extremely sensitive. 

### Translation errors

Minimal translation errors were identified. Occasionally, issues arose when English words in the source questionnaire could be translated in multiple ways, for instance, the word “okay” in an item asking participants to indicate their level of agreement with the statement “It is okay for a woman to have sex before marriage.” This item was correctly interpreted in English but there was ambiguity in how to translate it to other languages. Consequently, the final tool includes a note instructing translators to maintain its intended meaning of “alright” or “personally acceptable.”

## Discussion

Our findings suggest that a questionnaire exploring sexual practices, experiences and health-related outcomes can be comprehensible and acceptable by the general population in diverse global contexts, while highlighting the critical importance of rigorous processes for translation and cognitive testing of questionnaires intended for cross-cultural implementation. Through multiple waves of cognitive interviews in 19 countries, we identified several issues that made it difficult for participants to respond or led them to interpret draft items differently from intended. Iterative rounds of revision improved the alignment of items with measurement aims, reducing measurement error and bias, although these issues can never be completely removed.^11^ The minimal translation errors across many sites and languages underscores the strength of our rigorous translation approach.

The question–response problems we identified in our study were similar to the Cross National Error Source typology developed during the European Social Survey questionnaire design process.^18^ The typology classifies errors according to poor source question design; translation problems (resulting from either translator error or from source question design); and cultural portability. In our cognitive interviewing study, we distinguished an additional two sources of question failure – acceptability and knowledge barriers – because of the more sensitive nature of our research topic and the explicit aim of our study to explore the willingness of participants to respond. We were carefully attuned to the identification of knowledge barriers, because our study sample comprised both highly educated participants as well as those without a formal education and with low literacy across a diversity of ages.

Many of the items in our draft questionnaire were derived from pre-existing surveys, for example: Demographic and health surveys;^6^ National survey of sexual attitudes and lifestyle (Natsal-3 and −4);^19^ National survey of family growth;^20^ Australian study of health and relationships;^21^ Adolescent 360 survey;^22^ and Performance monitoring for action.^23^ The large number of pre-existing items that required revision highlights the critical importance of going beyond translation and perfunctory pretesting when adapting existing tools for a new setting. As others have found, rigorous cognitive testing of newly developed as well as previously validated questionnaire items can identify response errors when used in a new setting.^24^

Although we could improve most of the original items in the draft questionnaire, we had to remove a few items without replacement. In some instances, measures used in some contexts are not easily adaptable for cross-cultural comparative research but remain useful locally. For example, our findings concerning the item about ethnicity of most recent sexual partner align to those of another cross-cultural cognitive interviewing study that noted the poor cultural portability of an item on ethnicity,^25^ even though this item has been used successfully in some national sex surveys.^26^^,^^27^ In other instances, single items created confusion when dealing with layered constructs, such as experiences of discrimination based on sexual orientation or gender identity. Multi-item measures, which are beyond the scope of our questionnaire, would be better suited to assess these priority constructs.

A lack of study-specific funding resulted in a smaller number of interviews being conducted in Botswana and Uruguay before the wave 3 data collection period ended. Theoretical saturation had not been achieved in the Botswana site; we therefore used preliminary findings from Botswana in the global synthesis, but did not revise the questionnaire based on question failures that were identified only in this site. We recommend further cognitive testing and adaptation of the questionnaire before implementation in Botswana. 

In attempting to develop a basic questionnaire that is broadly applicable across diverse populations, there was inherent tension between revising items to be more acceptable in some settings and for some population groups and avoiding making items incomprehensible for others. Where specific revisions were only recommended by some study sites, we only amended the global questionnaire if such changes would not be outweighed by substantial decreases in comprehensibility across other sites. Occasionally, disagreements between study sites resulted in the development of adaptation notes (which accompany the final questionnaire) to provide guidance with specific items that require more extensive local adaptation before fielding.

No cognitive interviewing study can identify and mitigate all possible sources of error in a survey questionnaire. Although we developed our questionnaire through significant pretesting, further research is needed on how it performs when fielded in surveys. Because we employed concurrent probing during cognitive interviews, we cannot report expected survey completion times when not interrupted by probing. Because study participants were recruited purposively and agreed to participate in an interview discussing topics related to sex, we cannot comment on expected response rates when survey participants are selected randomly from a population. However, a separate study piloted an interim version of the questionnaire in a population-representative sample in Portugal during June–October 2023, and found reasonable completion times (average 18 minutes). A combination of web-based survey modality (70.9%; 1426/2010) and phone interview (29.1%; 584/2010) was used, resulting in 2010 completed questionnaires with response rates of 79.5% (web-based) and 12.4% (telephone) (Patrão AL and Nobre P, Faculty of Psychology and Education Sciences, University of Porto, Portugal, unpublished data, 2023).

Our questionnaire^10^ is intended to serve as a common core set of measures for research on sexual practices, experiences and health-related outcomes, and to be used either as a stand-alone module or integrated within broader sex- and/or health-related surveys. We suggest close monitoring and reporting on the performance of the questionnaire during its implementation in population-based survey research. We also encourage researchers implementing the questionnaire to use similar adaptation and translation approaches as used in this cognitive interviewing study, and to follow our implementation guidance in the careful adaptation of items containing terms with limited cultural portability.
